# Association of Brain‐Derived Neurotrophic Factor rs6265 G>A polymorphism and Post‐traumatic Stress Disorder susceptibility: A systematic review and meta‐analysis

**DOI:** 10.1002/brb3.2118

**Published:** 2021-04-09

**Authors:** Xi‐Yi Hu, Yu‐Long Wu, Chao‐Hui Cheng, Xiao‐Xi Liu, Lan Zhou

**Affiliations:** ^1^ Department of Mental Health Linyi Central Hospital Linyi China; ^2^ Department of Neurology Hubei Key Laboratory of Embryonic Stem Cell Research, Taihe Hospital Hubei University of Medicine Shiyan China

**Keywords:** brain‐derived neurotrophic factor, polymorphism, post‐traumatic stress disorder

## Abstract

**Background:**

Previous studies have shown that *the brain‐derived neurotrophic factor* (*BDNF*) rs6265 G > A polymorphism is closely related post‐traumatic stress disorder (PTSD) risk. However, the results were not consistent. We therefore conducted a meta‐analysis to explore the underlying relationships between *BDNF* rs6265 G > A polymorphism and PTSD risk.

**Materials and Methods:**

Five online databases were searched, and all related studies were reviewed up to July 1, 2020. Odds ratios (ORs) and corresponding 95% confidence intervals (CIs) were calculated to examine the statistical power of each genetic model. In addition, heterogeneity, sensitivity accumulative analysis, and publication bias were examined to check the statistical power.

**Result:**

Overall, 16 publications involving 5,369 subjects were included in this systematic review and 11 case‐control studies were analyses in meta‐analysis. The pooled results indicated an increasing risk of A allele mutations with PTSD risk. Moreover, the sequential subgroup analysis also demonstrated some similar situations in Asian populations and other groups.

**Conclusion:**

Current meta‐analysis suggests that the *BDNF* rs6265 G > A polymorphism might be involved in PTSD susceptibility.

## Graphical abstract

A meta‐analysis focused on the association between BDNF rs6265 G > A polymorphism and PTSD risk was conducted. 16 publications involving 5,369 subjects were included in this system review and 11 case‐controls were analyses in meta‐analysis. Current meta‐analysis suggests indicated that the BDNF rs6265 G > A polymorphism might be involved in PTSD susceptibility.

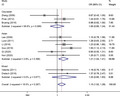

## 1 INTRODUCTION

Post‐traumatic stress disorder (PTSD) is a common and severe mental ailment characterized by behavioral, physiological, and hormonal alterations that occur after experiencing or witnessing a traumatic event and has a profound effect on patients’ lives and public health (Yehuda, 2002). In the USA, the reported prevalence of PTSD is 1%–14%, with an average of 8%; the lifetime prevalence of PTSD in women is approximately twice that in men (Breslau, 2001). With increasingly fierce competition and the growing number of sudden stressors in modern society, the etiological factors of PTSD have become more complex and the occurrence of PTSD is becoming more common (Kessler, 2000). Today, many risk factors have been confirmed to be related to PTSD, including trauma exposure, family history, individual characteristics, trauma history, previous behavioral or mental problems, and parental relationship characteristics (Keane et al., 2006; Stein et al., 2002).

In recent decades, there has been increasing evidence that PTSD is caused by interactions between various neural and traumatic factors (Disner et al., 2018; Joshi et al., 2020). In addition to the requisite etiological factor of trauma exposure for its onset, the abnormal expression of and functional changes in some neurotransmitters and neurotrophins, such as dopamine, serotonin, and brain‐derived neurotrophic factor (BDNF), are considered the most important factors that contribute to PTSD susceptibility (Miller et al., 2017; Rakofsky et al., 2012). BDNF is an important neurotrophic factor that participates in neuronal survival and growth‐promotion in the central nervous system, particularly in the hippocampus (Egan et al., 2003; Notaras & Buuse, 2020); alterations in BDNF levels are seen in the brain's fear circuit following trauma exposure (Burstein et al., 2018). BDNF expression, a potential biomarker for PTSD, is significantly lower in patients with PTSD than in healthy controls (Angelucci et al., 2014; Dell'Osso et al., 2009). However, a newly published meta‐analysis indicated that BDNF levels were significantly higher in the PTSD group than in healthy controls (Mojtabavi et al., 2020). Some studies have speculated that this increase is accompanied by acute restoration or reconstruction of brain neurons in the early stages after a traumatic experience (Hauck et al., 2010; Matsuoka et al., 2013). Animal‐based research has also found over‐expression of BDNF protein in the plasma and hippocampus of stressed rats relative to that in nonstressed controls in the compensatory stage (Faure et al., 2007; Zhang et al., 2014).

Rs6265 G > A is the most common single nucleotide polymorphism (SNP) locus in the 5′ promoter region of the BDNF gene, which is located on the short arm of chromosome 11p13. This gene variant involves a nucleotide substitution from guanine to adenine at position 196 in the BDNF coding region, resulting in a nonsynonymous amino acid alternation from valine (Val) to methionine (Met) in codon 66 of the BDNF prodomain (Egan et al., 2003). The Met allele exhibits abnormal intracellular trafficking and regulates the secretion of BDNF in comparison with the Val allele, which has always been suggested to be associated with lower BDNF release, resulting in a reduced release of activity‐dependent dopamine when neurons are activated (Egan et al., 2003). In terms of PTSD, Met allele carriers exhibit increased activity in neural structures and appear to be more susceptible to disease development (Lonsdorf et al., 2015). To date, the rs6265 G > A polymorphism has been shown to be associated with many central nervous system diseases, such as Alzheimer's disease, Parkinson's disease, depression, and suicide (Aldoghachi et al., 2019; Brown et al., 2020; Wang et al., 2015). In 2006, Zhang et al. conducted the first case–control study on the association between the BDNF rs6265 G > A polymorphism and PTSD susceptibility and found no significant association in a US population (Zhang et al., 2006). Since then, many studies have been published but the association between BDNF rs6265 G > A polymorphism and PTSD susceptibility remains controversial. Considering the inconsistencies among published studies, we conducted this meta‐analysis to further elucidate the association between BDNF rs6265 G > A polymorphism and PTSD susceptibility.

## 2 MATERIALS AND METHODS

This meta‐analysis was conducted according to the guidelines of the preferred reporting items for systematic reviews and meta‐analyses (PRISMA) statement (Moher et al., 2009). All collected information was obtained from all published articles, and no ethical approval was necessary.

### 2.1 Literature search

Three English databases (PubMed, Embase,and Web of Science), and two Chinese databases (CNKI and Wanfang) were used to identify studies on the association between BDNF rs6265 G > A polymorphism and PTSD susceptibility from database inception to July 1, 2020. The bibliographies of all included studies were reviewed to identify additional relevant studies. The strategy was listed (e.g., in PubMed):

#1 Brain Derived Neurotrophic Factor
#2 BDNF
#3 rs6265
#4 #1 OR #2 OR #3
#5 polymorphism
#6 variant
#7 mutation
#8 #5 OR #6 OR #7
#9 Post‐Traumatic Stress Disorder
#10 PTSD
#11 #9 OR #10
#12 #4 AND #8 AND #11

### 2.2 Inclusion and exclusion criteria

The following criteria were used to identify relevant studies: (a) only case‐control and cohort studies were selected; (b) studies on the association between BDNF rs6265 G > A polymorphism and PTSD susceptibility were selected, and subsequent meta‐analyses were conducted with studies in which the P value of the Hardy–Weinberg equilibrium (HWE) test in genotype distributions in the control group was greater than 0.05; (c) studies with sufficient genotype data for both case and control groups included; (d) studies published in English or Chinese; (e) subgroup analyses were conducted with at least two groups; and (f) studies with the latest or largest sample size were retained if multiple publications or overlapping data were found. The exclusion criteria included the following: (a) case report (case series), review articles; (b) biological fundamental and animal experiment studies; and (c) studies without sufficient genotype information.

### 2.3 Data extraction and quality evaluation

Two authors (Hu and Wu) independently reviewed all included studies and extracted the following information: the name of the first author, publication year, country and subject ethnicity, control design (healthy control or controls with traumatic exposures but without a PTSD diagnosis [PTSD^−^]), genotyping method, sample sizes of cases and controls, frequency information for the genotype distribution of the case and control groups, traumatic factors, assessment of the HWE in the control group, age distribution, and diagnostic criteria.

### 2.4 Statistical analysis

Crude odds ratios (ORs) and 95% confidence intervals (CIs) were calculated to examine the association between the BDNF rs6265 G > A polymorphism and PTSD susceptibility. Five genetic models of the rs6265 G > A polymorphism were examined: allele contrast (A versus. G), co‐dominant (GA versus. GG and AA versus. GG), dominant (GA + AA versus. GG), and recessive (AA versus. GG + GA). Heterogeneity among the included studies was examined using Cochran's Q and I^2^ tests (Huedo‐Medina et al., 2006). A random effects model was adopted when I^2^ > 40%; otherwise, a fixed effects model was adopted (DerSimonian, 1996; Mantel & Haenszel, 1959). All statistical analyses, including cumulative analysis, sensitivity analysis, publication biases, and subgroup analysis, were conducted with studies that satisfied the HWE criterion. Subgroup analyses were conducted based on differences in ethnicity, control design, traumatic factors, and sex differences. Cumulative meta‐analysis and sensitivity analysis were conducted to explore the tendency and verify the stability of results according to the shifting of dates. Potential publication biases were detected using Egger's linear regression test and Begg's funnel plots (Begg & Mazumdar, 1994; Egger et al., 1997). All statistical analyses were conducted using STATA version 14.0 (Stata Corporation, College Station, TX, USA). A value of *p* <.05 (two‐sided) was considered statistically significant.

## 3 RESULTS

### 3.1 Study characteristics

**FIGURE 1:**
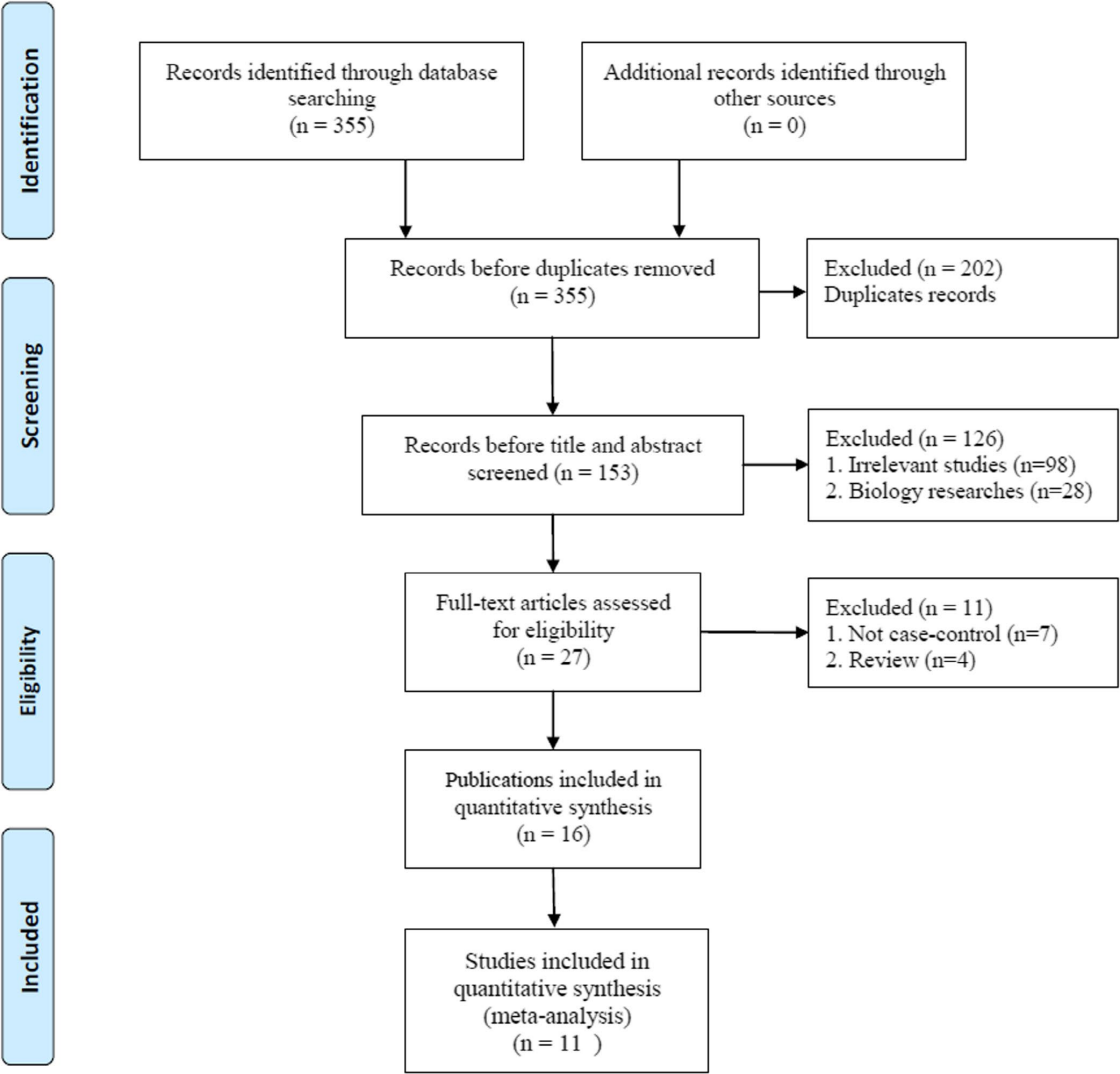
Flow diagram of the study selection process

The selection process is illustrated in Figure 1. First, 355 potential case–control studies were identified using a detailed search strategy. Second, 202 studies were deleted due to duplication or data overlap based on title and abstract screening. Third, 126 studies were excluded because of a lack of relevance or because they were fundamental biological studies following full‐text review. Fourth, 11 studies were excluded for two reasons: (a) seven studies were not case‐control studies and (b) four studies were reviewed. Finally, 16 studies with 1,739 patients and 3,630 controls met the inclusion and exclusion criteria (Bruenig et al., 2016; Dai et al., 2017; Dretsch et al., 2016; Guo et al., 2018, 2019; Heon‐Jeong et al., 2006; Hori et al., 2020; Jin et al., 2019; Li et al., 2016; Lyoo et al., 2011; Pivac et al., 2012; Qi et al., 2020; Valente et al., 2011; Young et al., 2018; Zhang et al., 2006, 2014). There were four studies in Caucasian populations (Bruenig et al., 2016; Pivac et al., 2012; Young et al., 2018; Zhang et al., 2006), nine studies in Asian populations (Dai et al., 2017; Guo et al., 2018, 2019; Heon‐Jeong et al., 2006; Hori et al., 2020; Jin et al., 2019; Li et al., 2016; Lyoo et al., 2011; Qi et al., 2020), and three studies in mixed populations (Dretsch et al., 2016; Valente et al., 2011; Zhang et al., 2014). Five studies used the polymerase chain reaction‐restriction fragment length polymorphism (PCR‐RFLP) method, while the rest used real‐time PCR, TaqMan, SnaPShot, Sequencer platform, and Illumina methods. According to the control source, the controls came from healthy populations in eight studies, and the controls experienced traumatic exposure but did not have PTSD symptoms in 10 studies. War combat, terrorist violence, natural disasters, and diseases were the most commonly reported sources of trauma (Table 1).

**TABLE 1:** Characteristics of case–control studies on BDNF rs6265 G > A polymorphisms and PTSD risk in system review

First author	Year	Country/Ethnicity	Control design	Genotyping method	Case	Control	Genotype distribution						*p* for HWE	Traumatic factors	Age distribution	Diagnostic criteria
							Case			Control						
							GG	GA	AA	GG	GA	AA				
Zhang	2006	US/Caucasian	HC	PCR‐RFLP	96	250	69	26	1	166	92	8	0.26	NA	Adult	DSM‐III
Lee	2006	Korea/Asian	HC	PCR‐RFLP	107	161	28	57	22	48	82	31	0.70	NA	Adult	DSM‐IV
Valente	2011	Brazil/Mixed	HC + PTSD^−^	Real‐time PCR	65	767	48	15	2	584	169	14	0.66	Urban violence	Adult	DSM‐IV
Lyoo	2011	Korea/Asian	HC	PRISM SnaPShot	30	36	10	15	5	14	18	4	0.62	Terroristattacks ‐fire	Adult	DSM‐IV
Pivac	2012	Croatia/Caucasian	PTSD^−^	TaqMan	370	206	234	124	12	140	63	3	0.17	Combat	Adult	DSM‐IV
Zhang	2014	US/Mixed	PTSD^−^	TaqMan	42	419	20	16	6	294	104	21	<0.01	Combat	Adult	NA
Li	2016	China/Asian	PTSD^−^	PCR‐RFLP	161	363	39	80	42	102	190	71	0.30	Earthquake	Adolescents	PCL‐C
Dretsch	2016	US/Mixed	PTSD^−^	PCR‐RFLP	41	185	28	8	5	129	49	7	0.39	Combat	Adult	PCL‐M
Bruenig	2016	Australia/Caucasian	PTSD^−^	Illumina	151	106	99	46	6	71	28	7	0.08	Combat	Adult	DSM V
Dai	2017	China/Asian	PTSD^−^	Sequenom MassARRAY iPLEX platform	28	167	2	26a		38	109a		NA	Flood	Adult	PCL‐C
Guo−1	2018	China/Asian	HC	sequenced by Sangon Biotech	300	150	94	119	87	45	52	53	<0.01	NA	Adult	DSM‐IV
Young	2018	US/Caucasian	PTSD^−^	Sequencer	58	168	36	22a		113	55a		NA	Combat	Adult	DSM‐IV
Jin	2019	Korea/Asian	HC	TaqMan	83	133	31	52a		36	97a		NA	Mixed	Adult	DSM V
Guo−2	2019	China/Asian	HC + PTSD^−^	PCR‐RFLP	102	298	14	59	29	88	143	67	0.54	Disease	Adult	DSM‐IV
Qi	2020	China/Asian	PTSD^−^	Ligase Detection technique	55	155	17	27	11	38	83	34	0.37	Lost Children	Adult	DSM‐IV
Hori	2020	Japan/Asian	HC	TaqMan	50	70	17	23	10	25	36	9	0.48	NA	Adult	PDS

Abbreviations: A, GA + AA genotype; DSM, Diagnostic and Statistical Manual of Mental Disorders; HC, Healthy control; HWE, Hardy–Weinberg equilibrium; MAF, Minor allele frequency in control group; NA, Not available; PCL‐C, PTSD Checklist Civilian Version; PCR‐RFLP, Polymerase chain reaction‐restriction fragment length polymorphism; PDS, Post‐traumatic Diagnostic Scale; PTSD, Post‐traumatic stress disorder; PTSD^−^, traumatic expose without PTSD symptom.

### 3.2 Meta‐analysis

**FIGURE 2:**
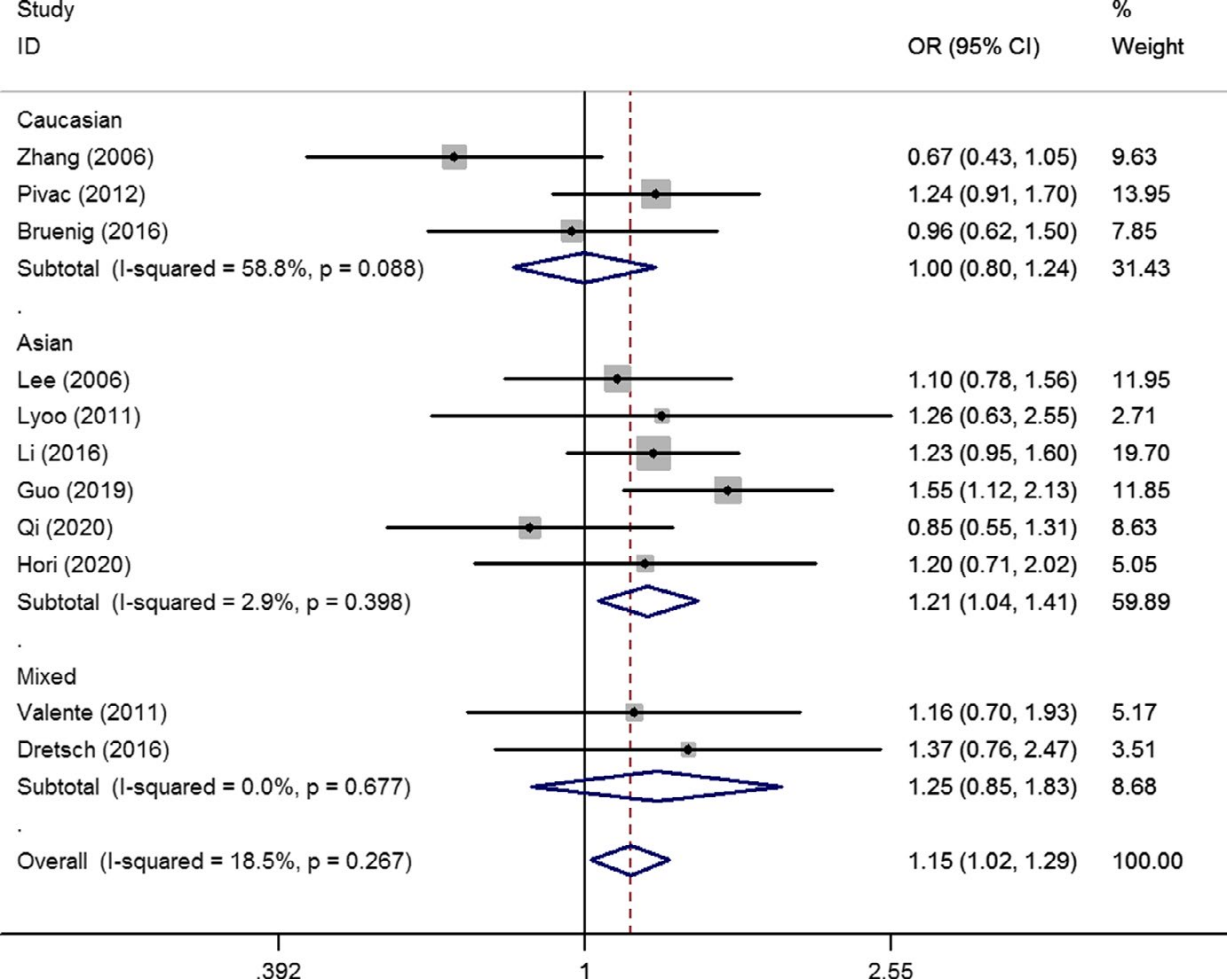
OR and 95% CIs of the associations between BDNF rs6265 G > A polymorphisms and PTSD risk in A versus. G model

After selecting studies according to their HWE status, two studies and three studies were removed because the P value of the HWE test was less than 0.05, or unavailable, respectively. Eleven studies involving 1,228 PTSD patients and 2,613 controls were included in the meta‐analysis (Bruenig et al., 2016; Dretsch et al., 2016; Guo et al., 2019; Heon‐Jeong et al., 2006; Hori et al., 2020; Li et al., 2016; Lyoo et al., 2011; Pivac et al., 2012; Qi et al., 2020; Valente et al., 2011; Zhang et al., 2006). The synthesized results demonstrated that the rs6265 G > A polymorphism significantly increased the risk of PTSD based on data from publications that satisfied the HWE conditions (A versus. G: OR = 1.15, 95% CI = 1.02–1.29, *p* =.02, *I* ^2^ = 18.5%; AA versus. GG: OR = 1.46, 95% CI = 1.11–1.92, *p* =.01, *I* ^2^ = 19.8%, (Figure 2); AA versus. GG + GA: OR = 1.30, 95% CI = 1.03–1.64, *p* =.03, *I* ^2^ = 0%) (Table 2). Subsequently, subgroup analysis based on differences in ethnicity revealed an increased PTSD risk in the Asian population (A versus. G: OR = 1.21, 95% CI = 1.04–1.41, *p* =.01, *I* ^2^ = 2.9%; AA versus. GG: OR = 1.52, 95% CI = 1.11–2.07, *p* =.01, *I* ^2^ = 11.8%; GA + AA versus. GG: OR = 1.30, 95% CI = 1.02–1.66, *p* =.03, *I* ^2^ = 39.6%; AA versus. GG + GA: OR = 1.30, 95% CI = 1.00–1.68, *p* =.05, *I* ^2^ = 0%) (Table 2), and mixed populations (AA versus. GG: OR = 2.56, 95% CI = 1.01–6.46, *p* =.05, *I* ^2^ = 0%; AA versus. GG + GA: OR = 2.64, 95% CI = 1.06–6.59, *p* =.04, *I* ^2^ = 0%) (Table 2). Moreover, the analyses based on control design indicated that the BDNF rs6265 G > A polymorphism significantly contributed to PTSD risk in the PTSD^−^ control groups (A versus. G: OR = 1.25, 95% CI = 1.02–1.54, *p* =.03, *I* ^2^ = 41.7%; AA versus. GG + GA: OR = 1.42, 95% CI = 1.07–1.89, *p* =.02, *I* ^2^ = 16.8%). A similar increased risk of rs6265 G > A mutation in patients with PTSD risk was also observed following natural disasters and diseases exposure (A versus. G: OR = 1.35, 95% CI = 1.10–1.66, *p* <.01, *I* ^2^ = 14.6%; AA versus. GG: OR = 1.90, 95% CI = 1.25–2.90, *p* <.01, *I* ^2^ = 35.5%; AA versus. GG + GA: OR = 1.42, 95% CI = 1.02–1.97, *p* =.01, *I* ^2^ = 0%) (Table 2).

**TABLE 2:** Summary ORs and 95% CI of BDNF rs6265 G > A polymorphisms and PTSD risk in studies with HWE

Locus	N*	A versus. G				GA versus. GG				AA versus. GG				GA + AA versus. GG				AA versus. GG + GA			
		OR	95% CI	*p*	*I* ^2^ (%)^a^	OR	95% CI	*p*	*I* ^2^ (%)^a^	OR	95% CI	*p*	*I* ^2^ (%)^a^	OR	95% CI	*p*	*I* ^2^ (%)^a^	OR	95% CI	*p*	*I* ^2^ (%)^a^
Total	11	1.15	1.02–1.29	0.02	18.5	1.10	0.93–1.30	0.28	21.1	1.46	1.11–1.92	0.01	19.8	1.15	0.97–1.35	0.10	28.4	1.30	1.03–1.64	0.03	0
Ethnicity																					
Caucasian	3	1.00	0.80–1.24	0.98	58.8	1.02	0.78–1.32	0.91	37.4	0.88	0.29–2.72	0.83	46.1	0.98	0.67–1.43	0.90	51.2	0.87	0.30–2.55	0.80	41.5
Asian	6	1.21	1.04–1.41	0.01	2.9	1.23	0.95–1.59	0.12	37.1	1.52	1.11–2.07	0.01	11.8	1.30	1.02–1.66	0.03	39.6	1.30	1.00–1.68	0.05	0
Mixed	2	1.25	0.85–1.83	0.26	0	0.95	0.58–1.56	0.84	0	2.56	1.01–6.46	0.05	0	1.11	0.70–1.74	0.66	0	2.64	1.06–6.59	0.04	0
Control design																					
HC	6	1.01	0.92–1.31	0.31	22.2	1.11	0.78–1.59	0.56	40.1	1.46	0.96–2.20	0.08	0	1.14	0.80–1.63	0.47	45.0	1.17	0.82–1.66	0.38	0
PTSD^−^	7	1.25	1.02–1.54	0.03	41.7	1.23	0.90–1.67	0.19	43.1	1.66	0.97–2.82	0.06	48.2	1.30	0.96–1.17	0.09	48.0	1.42	1.07–1.89	0.02	16.8
Traumatic source																					
Other	4	0.94	0.76–1.16	0.54	25.9	0.85	0.62–1.16	0.31	0	0.99	0.62–1.59	0.94	0	0.86	0.64–1.16	0.33	4.9	1.03	0.68–1.56	0.87	0
Violence	2	1.20	0.79–1.81	0.39	0	1.10	0.65–1.86	0.72	0	1.74	0.59–5.16	0.32	0	1.16	0.71–1.92	0.55	0	1.64	0.58–4.65	0.35	0
War	3	1.17	0.93–1.49	0.08	0	1.12	0.84–1.49	0.46	0	1.65	0.58–4.68	0.35	55.3	1.16	0.88–1.53	0.29	0	1.63	0.54–4.91	0.38	60.5
E + D	2	1.35	1.10–1.66	<0.01	14.6	1.64	0.71–3.79	0.25	78.3	1.90	1.25–2.90	<0.01	35.5	1.74	0.82–3.68	0.15	75.3	1.42	1.02–1.97	0.04	0
Sex differences																					
Female	3	1.24	0.89–1.74	0.20	0	1.05	0.61–1.83	0.85	0	1.61	0.82–3.18	0.17	0	1.25	0.86–1.82	0.24	0	1.53	0.86–2.72	0.15	0
Male	4	1.11	0.88–1.40	0.39	0	1.18	0.88–1.58	0.27	0	1.05	0.52–2.09	0.89	27.1	1.15	0.89–1.50	0.29	0	0.96	0.49–1.87	0.90	31.1

Abbreviations: E + D, Earthquake and disease; HC, Healthy control; HWE, Hardy–Weinberg equilibrium; PTSD, Post‐traumatic stress disorder; PTSD^−^, traumatic expose without PTSD symptom.

### 3.3 Sensitivity and accumulative analysis

**FIGURE 3:**
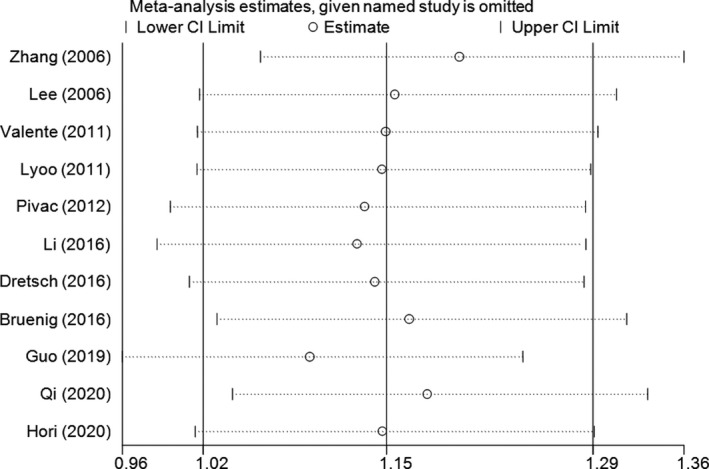
Sensitivity analysis involving deletion of each study to reflect the influence of the individual dataset to the pooled ORs in A versus. G model of BDNF rs6265 G > A polymorphism

**FIGURE 4:**
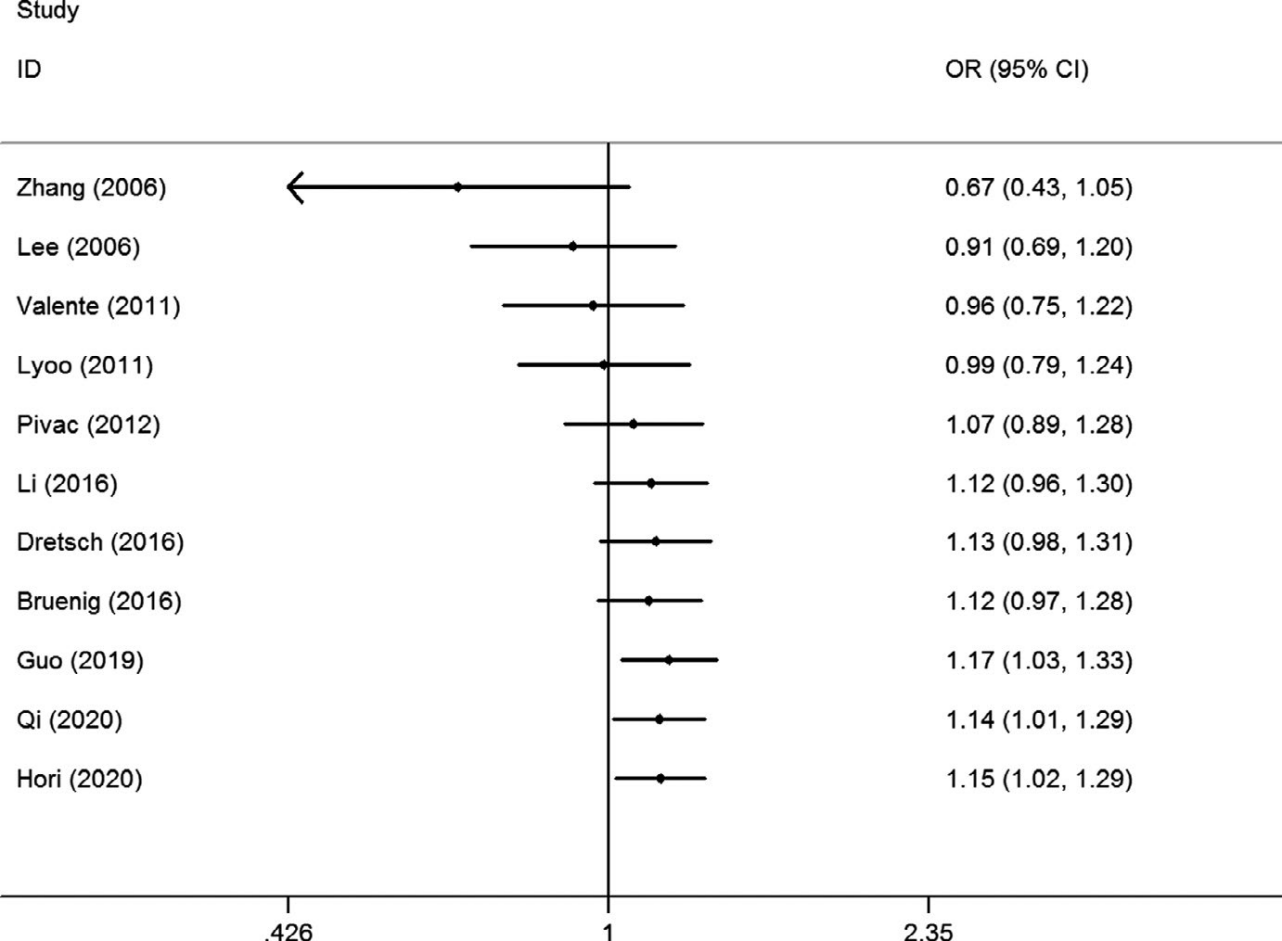
Cumulative meta‐analyses according to publication year in A versus. G model of BDNF rs6265 G > A polymorphism

Sensitivity analysis was conducted by removing each study one by one according to the publication date; the results demonstrated some slight fluctuations after excluding the studies of Pivac et al. (OR = 1.13, 95% CI = 1.00–1.29), Li Guo et al. (OR = 1.13, 95% CI = 0.99–1.29), and Guo et al. (OR = 1.09, 95% CI = 0.96–1.24) (Figure 3 for A versus. G model). Accumulative analysis was also performed and showed a progressively increasing effect on PTSD risk (Figure 4 for A versus. G model).

### 3.4 Publication bias

**FIGURE 5:**
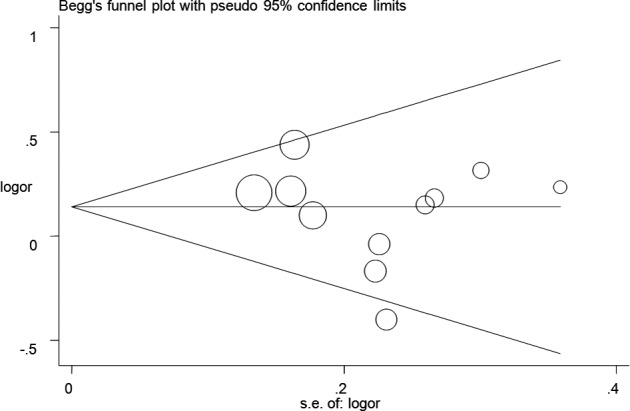
Funnel plot analysis to detect publication bias for A versus. G model of BDNF rs6265 G > A polymorphism. Circles represent the weight of the studies

Publication bias was evaluated, and funnel plots did not demonstrate any significant asymmetry (Figure 5 for A versus. G model). The results were confirmed using Egger's test (A versus. G, *p* =.38; GA versus. GG: *p* =.77; AA versus. GG, *p* =.59; GA + AA versus. GG, *p* =.94; AA versus. GG + GA, *p* =.91).

## 4 DISCUSSION

PTSD is a serious disorder that occurs after experiencing unusual psychological trauma, such as plague, natural disasters, violent events, and war (Charlson et al., 2019). It is generally divided into three categories: (a) “re‐experiencing symptoms,” in which these traumatic events will appear repeatedly in the mind, such as in dreams and in involuntary thoughts; (b) “avoidance phenomenon,” which includes avoiding going back to or talking about traumatic events and becoming numb; (c) “arousal and reactivity symptoms,” in which it is easier to have a strong response to external stimuli; and (d) “cognitive and mood‐related symptoms,” which include negative thoughts about oneself or the world and loss of interest in positive activities or emotions. PTSD is more common in women than in men, and sex differences may be an important factor influencing PTSD susceptibility. First, abnormal changes in hormone levels, especially estrogen levels, can increase sensitivity to traumatic stimuli in female patients and involved in and affect neurobiological systems associated with PTSD (Christiansen & Berke, 2020). For female individuals, estrogen and progesterone contribute to cognitive‐emotional processes in PTSD (Maddox et al., 2018), and high levels of estradiol have negative effects on the response to traumatic stress (Albert et al., 2015). Second, women, like children, are more likely to be victims of all kinds of violence and sexual abuse than men (Birkeland et al., 2017; Catabay et al., 2019). It is worth noting that the current results of the subgroup analysis of sex differences did not find any significant difference in the genotype distribution between the female and male groups. This inconsistency might be due to the limited number of studies and participants; these results still need to be confirmed in the future.

Many studies have shown that the occurrence of PTSD is often accompanied by damage to brain tissue, which in turn leads to neuronal cell dysfunction (Nampiaparampil, 2008). BDNF is distributed across multiple brain regions and plays a key role in neurophysiological processes, such as neuroprotection, maturation, repair, and maintenance of neurons. Several studies have shown that the expression levels and protein activity of BDNF are important for neurophysiological regulation (Ji et al., 2015). BDNF production and activity can be genetically determined and controlled using mutated sequence regions in its gene. In mammalian, BDNF is critically involved in synaptic plasticity and is implicated in hippocampus‐dependent learning and memory (Bramham & Messaoudi, 2005; Hariri et al., 2003); memory abnormalities are considered a core feature of PTSD, and patients with PTSD always present with a negative memory bias relative to healthy controls (Itoh et al., 2019). The abnormal involuntary recovery of traumatic memories, including invasive thoughts, flashbacks, and nightmares, often causes great mental pain. Recent studies have indicated that this BDNF polymorphism could dramatically alter the intracellular trafficking and packaging of pro‐BDNF, subsequently regulating the secretion of mature peptides (Egan et al., 2003). Individuals with the Met allele always present with a lower level of hippocampal N‐acetyl aspartate compared to those with the Val allele. In knock‐in mice, extinction learning was impaired in Met allele carriers compared to non‐Met allele carriers (Soliman et al., 2010); there was a similar finding in patients with PTSD with the Met allele when compared to those with the Val allele (Felmingham et al., 2018). A human study by Horri et al. revealed that PTSD patients with the Met allele had significantly worse memory performance than controls, indicating that the rs6265 polymorphism could be involved more in core memory abnormalities than general memory dysfunction in PTSD (Hori et al., 2020).

To date, the rs6265 G > A locus has been suggested to correlate with a notably lower serum level, and negative effects that alter the signal transduction pathway, resulting in a close relationship with the occurrence and prognosis of PTSD. In 2006, Zhang et al. conducted the first case‐control study in the US population and found no significant association between the BDNF gene variant (rs6265 G > A) and PTSD (Zhang et al., 2006). Subsequent studies have been conducted to examine the association between the BDNF polymorphic locus and PTSD susceptibility and have produced inconsistent and confusing results. In 2012, Pivac et al. reported an increased risk of PTSD with the BDNF A allele in Caucasian veterans (Pivac et al., 2012). Moreover, Li et al., Dretsch et al., and other researchers also reported an elevated risk of PTSD (Dretsch et al., 2016; Li et al., 2016). In contrast, Jin et al. found that the GG genotype may play a critical role in the occurrence of PTSD (Jin et al., 2019). In addition, Bruenig et al. and others did not find any significant association between the BDNF s6265 G > A polymorphism and PTSD risk (Bruenig et al., 2016).

How can we reach a more precise conclusion regarding the relationship between BDNF rs6265 G > A polymorphism and PTSD risk with these current inconsistent results? To the best of our knowledge, meta‐analysis is the most valuable method for resolving the current confusion due to the shortage of samples. In this meta‐analysis, we examined the correlation between the BDNF rs6265 G > A polymorphism and PTSD susceptibility, based on 11 publications that met the inclusion criteria. In our meta‐analysis, we comprehensively summarized the current evidence regarding the association between BDNF rs6265 G > A polymorphism and PTSD susceptibility. According to the pooled data, there was a significant correlation between the A mutation and an increased risk of PTSD. Subsequently, a subgroup analysis was conducted and a similar elevated risk was observed with this variant of PTSD susceptibility, especially in the Asian population and PTSD^−^ groups. Our results indicate that that the mutation from G to A plays an even more active role in some Asians, which might be due to a large sample of Asian individuals included in the integrated studies. In terms of study design, the patients with of BDNF rs6265 G > A polymorphism presented a significantly higher risk than the PTSD^−^ groups. This polymorphism locus may play an active role in the traumatic factors when the case and PTSD^−^ groups were faced with the same exposures, which also indicates that the traumatic event plays a triggering role in the etiology of PTSD development through the interactions of genetic and environmental factors.

In 2016, Bruenig et al. published a meta‐analysis on BDNF rs6265 G > A polymorphism and PTSD susceptibility and suggested a potential protective factor for the GG genotype. The results were based on nine case‐control studies, including those that deviated from the HWE conditions (Bruenig et al., 2016). In 2017, Bountress et al. conducted another meta‐analysis and their results indicated a marginally significant effect of the Met allele on increasing PTSD risk (OR = 1.20; 95% CI = 0.99–1.26; *p* =.057). In their meta‐analysis, nine studies were included, but the results obtained were based on different genetic models. Moreover, only one genetic model was examined to determine the association between BDNF rs6265 G > A polymorphism and PTSD susceptibility, without any subgroup analysis or quantitative assessment (Bountress et al., 2017). Therefore, we conducted this meta‐analysis to gain a better insight into the trends in the results from earlier publications.

To our knowledge, this systematic review and meta‐analysis included all current publications that assessed the association between the BDNF rs6265 G > A polymorphism and PTSD susceptibility. There were some limitations to this study. First, the quantitative analysis was conducted with only 11 publications; the other studies were eliminated because of a lack of data or because the P value of the genotype distribution deviated from the HWE. Therefore, the inference based on a small sample size might be biased, leading to deviation in the pooled results. Second, the BDNF rs6265 G > A polymorphism was the only locus examined in this meta‐analysis, and the interactive effects of different SNPs and other environmental or lifestyle factors were not assessed simultaneously. Third, most studies were based on Caucasian or Asian participants, and the current results may not be applicable to all populations. Finally, only reports published in Chinese or English were included in this meta‐analysis, which may have resulted in a language bias. Despite these shortcomings, some positive aspects were found to enhance the quality of our study: (a) More studies were included than in previous analyses; (b) all five genetic models were examined in general and subgroups to explore the potential relationship between rs6265 G > A polymorphism and PTSD; (c) the statistics examined were taken from studies that satisfied the HWE for the genotype distribution in controls; (d) no significant heterogeneity was found, indicating a fair consistency among all included studies; and (e) no significant publication bias was found using Egger's test and Begg's funnel plots.

## 5 CONCLUSION

Our results from this meta‐analysis suggest that the BDNF rs6265 G > A polymorphism is associated with PTSD susceptibility in Asian people. Further studies are needed to assess the association between this SNP and PTSD.

## CONFLICTS OF INTEREST

The authors declare no competing financial interests.

## AUTHOR CONTRIBUTION

HXY, WYL, LXX, and ZL conceived the study. HXY and WYL searched the databases and extracted the data. HXY and CCH analyzed the data. HXY, LXX, and ZL wrote the draft of the paper. All the authors approved the final manuscript.

## ETHICAL APPROVAL

All collected information was obtained from all published articles, and no ethical approval was necessary.

### PEER REVIEW

The peer review history for this article is available at https://publons.com/publon/10.1002/brb3.2118.

## Data Availability

All data collected or analyzed for this study are available from the corresponding author upon reasonable request to any researchers.
